# Mesenchymal Stem Cells Secrete Multiple Cytokines That Promote Angiogenesis and Have Contrasting Effects on Chemotaxis and Apoptosis

**DOI:** 10.1371/journal.pone.0035685

**Published:** 2012-04-25

**Authors:** Robert A. Boomsma, David L. Geenen

**Affiliations:** 1 Department of Biology, Trinity Christian College, Palos Heights, Illinois, United States of America; 2 Section of Cardiology, Department of Medicine, University of Illinois at Chicago, Chicago, Illinois, United States of America; German Cancer Research Center, Germany

## Abstract

We have previously shown that mesenchymal stem cells (MSC) improve function upon integration in ischemic myocardium. We examined whether specific cytokines and growth factors produced by MSCs are able to affect angiogenesis, cellular migration and apoptosis. Conditioned media (CM) was prepared by culturing MSC for 48 hours. CM displayed significantly elevated levels of VEGF, Monocyte Chemoattractant Protein-1 (MCP-1), macrophage inflammatory protein-1α (MIP-1α), MIP-1β and monokine induced by IFN-γ (MIG) compared to control media. MSC contained RNA for these factors as detected by RT-PCR. CM was able to induce angiogenesis in canine vascular endothelial cells. MCP-1 and MIP-1α increased cell migration of MSC while VEGF reduced it. H9c2 cells treated with CM under hypoxic conditions for 24 hours displayed a 16% reduction in caspase-3 activity compared to controls. PI 3-kinase γ inhibitor had no effect on controls but reversed the effect of CM on caspase-3 activity. MCP-1 alone mimicked the protective effect of CM while the PI 3-Kγ inhibitor did not reverse the effect of MCP-1. CM reduced phospho-BAD (Ser112) and phospho-Akt (Ser473) while increasing phospho-Akt (Thr308). MCP-1 reduced the level of phospho-Akt (Ser473) while having no effect on the other two; the PI 3-Kγ inhibitor did not alter the MCP-1 effect. ERK 1/2 phosphorylation was reduced in CM treated H9c2 cells, and inhibition of ERK 1/2 reduced the phosphorylation of Akt (Ser473), Akt (Thr308) and Bad (Ser112). In conclusion, MSC synthesize and secrete multiple paracrine factors that are able to affect MSC migration, promote angiogenesis and reduce apoptosis. While both MCP-1 and PI3-kinase are involved in the protective effect, they are independent of each other. It is likely that multiple pro-survival factors in addition to MCP-1 are secreted by MSC which act on divergent intracellular signaling pathways.

## Introduction

Ischemic injury to the heart results in scarring of the myocardium and significant reduction in function. Numerous studies have been performed to determine the potential for exogenous stem cells to reverse the deleterious effects of myocardial infarction, and the results have all shown an improvement in cardiac function. However, the mechanism leading to this improvement is unclear, since not all investigators have seen a reduction in scarring due to cardiomyocyte differentiation of stem cells.

We have recently shown that intravenously injected mesenchymal stem cells (MSC) are able to prevent the loss of function that occurs in mouse hearts following permanent coronary artery occlusion [1]. This beneficial effect is seen without any reduction in scar tissue and ventricular dilatation or significant levels of stem cell differentiation into cardiomyocytes. It may be that stem cells are able to contribute to functional improvements by mechanisms such as increased blood vessel formation or a reduction in cell death [2]. In order to test this hypothesis we monitored the production of cytokines by MSC. We then determined the effect of MSC paracrine factors on cellular migration of MSC, angiogenesis by canine vascular endothelial cells and apoptosis in H9c2 myoblasts.

## Materials and Methods

Mice were housed in the Biological Resources Laboratory at UIC (AAALAC accredited) and maintained in accordance with the *Guide for the Care and Use of Laboratory Animals* (National Research Council, revised 1996). Experimental protocols were approved by the Institutional Animal Care and Use Committee at UIC.

### Mesenchymal Stem Cell (MSC) Culture

Mesenchymal stem cells (MSC) were isolated from FVB.Cg-Tg(GFPu)5Nagy/J mice (Jackson Laboratory) as described previously. Briefly, bone marrow cells were enriched for lineage negative (Lin−) cells using the SpinSep system (Stem Cell Technologies) and plated on tissue culture treated plates at a density of 0.1×10^6^ cells/cm^2^ in murine Mesencult media (basal media + stimulatory supplement; Stem Cell Technologies) with 100 units/ml penicillin, 100 µg/ml streptomycin and 0.25 µg/ml amphotericin B added. MSC from passage 15–20 were used in these studies which were found to be Sca-1^+^, CD34^+^, CD45^low^, CD90.1^−^, CD105^−^, and cKit^−^ as determined by flow cytometry [1].

MSC-conditioned media (CM) was prepared by plating 2.0×10^6^ MSC in 10 ml Mesencult on 100 mm tissue culture treated plates for 48 hours. Following culture, the media was filtered through a 0.22 µm membrane.

### Analysis of MSC for Growth Factors and Cytokines

In order to determine whether MSC secrete paracrine factors, Mesencult and CM were analyzed for the presence of various growth factors and cytokines using multiplex assay kits and Luminex technology (BioSource/Invitrogen). A custom mouse 10-plex kit was designed to test for vascular endothelial growth factor (VEGF), monocyte chemoattractant protein-1 (MCP-1), interferon-γ (IFN-γ), monokine induced by IFN-γ (MIG), macrophage inflammatory protein-1α (MIP-1α), MIP-1β, MIP-3β, tumor necrosis factor-α (TNF-α), fibroblast growth factor-basic (FGF-basic), and regulated upon activation normal T-cell expressed and secreted (RANTES). A mouse single-plex kit was used for platelet derived growth factor-BB (PDGF-BB). All procedures were carried out according to the manufacturer's instructions. Briefly, 50 µl of media was added to wells containing antibody coated beads and incubated 2 hours at room temperature. The beads were washed, treated with biotinylated detector antibody for 1 hour, washed, and incubated with streptavidin-RPE for 30 minutes. Following washing, fluorescence was detected using a Luminex-100 instrument and analyzed with proprietary software.

In order to determine whether MSC synthesized the paracrine factors identified in CM, RNA was isolated directly from MSC cultures grown two days in Mesencult in 6-well plates using an RNA4PCR kit including DNAse treatment (Ambion). Reverse transcription of 1 µg RNA was performed using an iScript cDNA Synthesis Kit with blended oligo(dT) and random hexamer primers (BioRad). PCR was done using iTaq DNA polymerase (BioRad), 1.5 mM MgCl_2_, 200 µM dNTP, 2 µl RT reaction product and 300 µM primers (Table 1). After an initial 95°C hot start, 30–50 cycles of 95°C (30 seconds), 55°C (30 seconds) and 72°C (1 minute) was performed.

**Table 1:** PCR Primers.

	Forward	Reverse
**VEGF**	CAGGCTGCTGTAACGATGAA	TGTCTTTCTTTGGTCTGCATTC
**MCP-1**	AGGTCCCTGTCATGCTTCTG	TCTGGACCCATTCCTTCTTG
**MIG**	TCTTTTCCTCTTGGGCATCATCTT	TTTCCCCCTCTTTTGCTTTTTCTT
**MIP-1α**	TGCCCTTGCTGTTCTTCTCT	CCCAGGTCTCTTTGGAGTCA
**MIP-1β**	TGTCTGCCCTCTCTCTCCTC	GTCTGCCTCTTTTGGTCAGG

### Angiogenesis Assay

The effect of paracrine factors secreted by MSC into CM on angiogenesis was studied using canine jugular vein vascular endothelial cells (CVEC; AllCells) and the Fibrin Gel In Vitro Angiogenesis Assay Kit (Chemicon #ECM630). CVEC were initially cultured on gelatin-coated plates in CVEC basal medium plus stimulatory supplements (AllCells). Adherent cells were removed with trypsin and placed in culture wells coated with a fibrin matrix as described by the manufacturer (Chemicon). 50,000 CVEC/well in 24-well plates or 5,000 cells/well in 96 well plates were plated on the fibrin matrix in one of three types of media (n = 19): 1) Mesencult (negative control), 2) Mesencult +10 mg/ml insulin, 5.5 mg/ml transferrin, 5 ng/ml sodium selenite (positive control) or 3) conditioned media (CM). The cells were covered 24 hours later with a second layer of fibrin, fresh media was applied, and the cells were cultured for an additional 4 days. Cultures were analyzed for the presence of endothelial capillary tube cellular networks using a semiquantitative scale (0–4) where 0 = cultures with no cellular organization and 4 = cultures where cells showed complete organization with polygonal structure.

### Cell Migration Assay

The effect of VEGF, MCP-1 and MIP-1α on MSC migration was studied by seeding 600,000 cells in 500 µl Mesencult onto 10 mm diameter tissue culture inserts with a 3.0 µM pore polycarbonate membrane (Nunc). The inserts were placed in 24-well culture plates containing 600 µl/well Mesencult with or without 30 ng/ml VEGF, 30 ng/ml MCP-1 or 100 pg/ml MIP-1α. After 6 hours at 37°C, the membranes were stained for 5 min in 30 µM acridine orange, washed in PBS, cut out, and mounted on slides in PBS with a coverslip. The underside of the membrane was viewed on a fluorescence microscope (490 nm excitation/520 nm emission), and the number of yellow-green fluorescing nuclei counted using the 20× objective. A mean of ten random visual fields was measured per membrane and each membrane was considered one trial. Data were expressed as a percent of control and analyzed using Student's t-test with p<0.05 considered statistically significant (n = 6).

### Caspase-3 Assay

The effect of paracrine factors secreted by MSC on caspase-3 was studied by treating rat embryonic cardiac myoblast H9c2 cells (ATCC #CRL-1446) with CM. 1.5×10^6^ H9c2 cells in 10 ml DMEM +10% FBS were plated on 100 mm tissue culture treated plates. After 24 hours the media was changed to DMEM +1% heat inactivated horse serum and the cells were cultured for an additional 48 hours. The media was replaced with Mesencult (control) or CM and the cultures were incubated under hypoxic conditions (1% O_2_, 5% CO_2_, 94% N_2_) for up to 24 hours. In some cases, either 1 µM phosphatidylinositol 3-kinase γ (PI 3-Kγ) inhibitor (Calbiochem #528106) in DMSO or 4 nM mouse recombinant MCP-1 (GenWay #10-783-79110) were added to the cultures. Equal amounts (1%) of DMSO (vehicle) were added to control cultures when the PI 3-Kγ inhibitor was used.

Caspase-3 activity in cell lysates was determined using an enzymatic assay. H9c2 cells treated with Mesencult, CM, MCP-1 or PI 3-Kγ inhibitor for 24 hours were lifted with trypsin (including all cells floating in media and washes) and 1–2×10^6^ cells were analyzed for caspase-3 activity using the ApoAlert Caspase-3 Colorimetric Assay Kit (Clontech #630216). For each experimental run, the cell number for each treatment group was equalized. Cells were lysed by incubating on ice for 10 minutes with ice cold lysis buffer. Cells were centrifuged at 14,000×g for 5 min. and the supernatant was assayed according to the manufacturer's directions. Caspase-3 activity was expressed as a percent of control activity and analyzed using Student's t-test.

### ELISA Assay

An ELISA was used to detect changes in the levels of total-Bad, phospho-Bad (Ser112), total-Akt, phospho-Akt (Ser473), phospho-Akt (Thr308) and ERK 1/2 using PathScan Sandwich ELISA Kits (Cell Signaling Technology #7162, 7182, 7170, 7160, 7252) or a STAR ERK 1/2 ELISA kit (Upstate #17-463). H9c2 cultures were treated with Mesencult, CM, MCP-1, 1 µM PI 3-Kγ inhibitor or 30 µM ERK 1/2 inhibitor (ERK inhibitor II; Calbiochem) for 6 or 24 hours and analyzed. Cell lysates were prepared by incubating H9c2 cultures (100 mm plates) on ice for 10 minutes with 500 µl ice cold lysis buffer (Cell Signaling Technology #9803)+1 mM PMSF. Cells were scraped, sonicated, centrifuged at 14,000×g for 5 min., and the supernatant stored at −80°C. Lysates were tested according to the manufacturer's instructions and analyzed with a plate reader at 450 nm. Protein concentration in cell lysates was determined using a BCA protein assay kit (Pierce). Levels were calculated per mg protein, expressed as a percent of the relevant control, and analyzed using Student's t-test.

## Results

### Growth Factors and Cytokines in MSC-Conditioned Media (CM)

**Figure 1:**
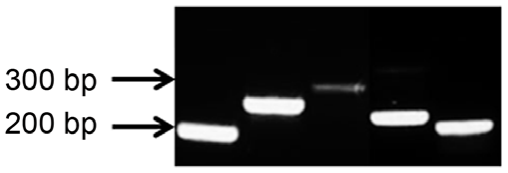
PCR products from RT reactions on RNA isolated from MSC. From left to right: VEGF, MCP-1, MIG, MIP-1α, MIP-1β.

Levels of growth factors and cytokines were measured in Mesencult (control) media (n = 3) and CM (n = 5) using Luminex technology. VEGF, MCP-1, MIG, MIP-1α and MIP-1β were absent in control media and found to be elevated in CM (Table 2). RNA transcripts for these five growth factors and cytokines were detected in MSC using RT-PCR (Figure 1). MIP-3β, IFN-γ, TNF-α, and PDGF-BB were absent in both types of media while FGF-basic and RANTES were present in very low concentrations in both control media and CM (data not shown).

**Table 2:** Cytokines in MSC-Conditioned Media.

	VEGF	MCP-1	MIG	MIP-1α	MIP-1β
**Mes (n = 3)**	ND	ND	ND	ND	ND
**CM (n = 5)**	4279±187	3901±255	11.48±0.79	9.41±3.88	57.37±3.72

Data reported as mean ± SE in pg/ml. Mes = Mesencult, CM = MSC-conditioned media, ND = non-detectable.

### Effect of MSC-Conditioned Media on Angiogenesis in CVEC

**Figure 2:**
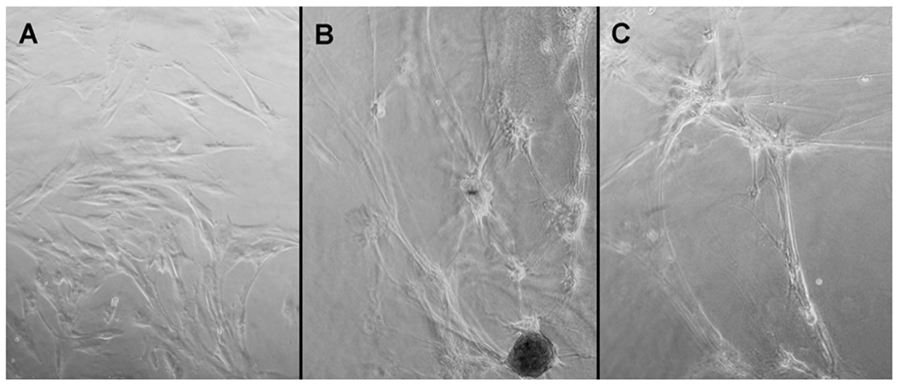
Effect of MSC-conditioned media on angiogenesis. Canine vascular endothelial cells plated on a fibrin matrix exposed to treatment media for 5 days. A: Cells treated with Mesencult. B: Cells treated with Mesencult containing insulin, transferrin and sodium selenite. C: Cells treated with conditioned media.

To determine whether the paracrine factors released by MSC promote angiogenesis, canine vascular endothelial cells (CVEC) were cultured on a fibrin matrix in the presence or absence of CM (Figure 2). In every case (n = 19), CVEC cultures treated with CM (Figure 2C) developed complex capillary networks (semiquantitative scores of 2–3) similar to those observed in the positive control cultures containing insulin, transferrin and sodium selenite (Figure 2B; scores = 3–4). These networks were absent in control cultures (Figure 2A; scores = 0–1).

### Effect of Cytokines on MSC Migration

**Figure 3:**
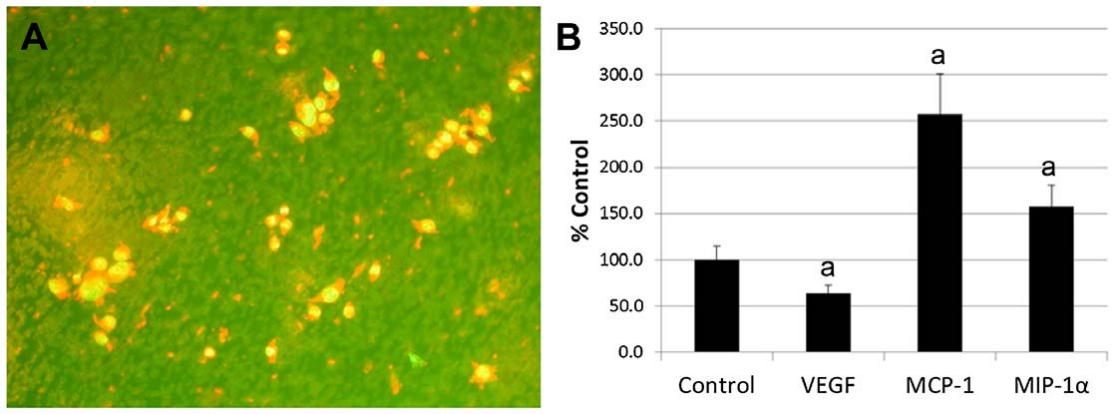
Effect of cytokines on MSC migration. A: Cells stained with acridine orange on the underside of the 3 µm polycarbonate membrane after MIP-1α treatment. Yellow-green = DNA; red = RNA. B: Effect of VEGF, MCP-1 and MIP-1α on MSC migration. Data expressed as a mean percent of Mesencult (control) treated cultures ± SE (n = 6). a = p<0.05 compared to controls.

The cytokines secreted by MSC were tested for their ability to affect the migration of MSC through a 3.0 µM polycarbonate membrane (n = 6). Cells were counted when the nuclei were observed on the underside of the membrane (Figure 3A). As shown in Figure 3B, 30 ng/ml VEGF treatment resulted in a decrease in MSC migration (63.8±10.9% of control) while 30 ng/ml MCP-1 and 100 pg/ml MIP-1α increased migration to 257.1±43.7% and 157.6±23.2% of control, respectively (p<0.05). Since the VEGF mediated reduction in MSC migration was surprising, we tested 3 ng/ml VEGF and found that this concentration also reduced migration (50.1±7.2% of controls, p<0.01).

### Effect of MSC-Conditioned Media on Caspase-3 and Akt/Bad Phosphorylation in H9c2 Cells

**Figure 4:**
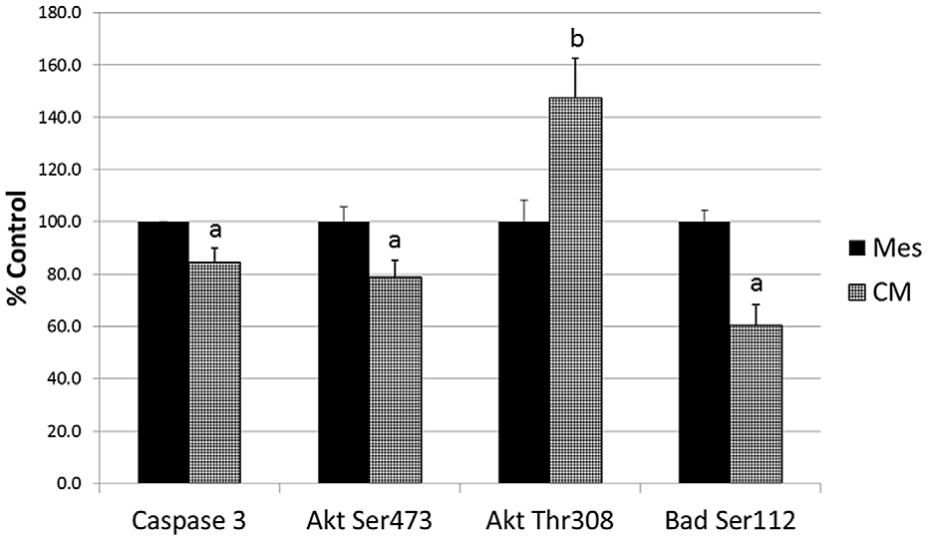
Effect of MSC-conditioned media on caspase-3, Akt and Bad. Changes in caspase-3, phospho-Akt (Ser473), phospho-Akt (Thr308) and phospho-Bad (Ser112) in H9c2 cells treated with Mesencult (Mes) or conditioned media (CM) for 24 hours under hypoxic conditions. Data calculated as a mean percent of Mesencult (control) treated cultures ± SE. a = p<0.05, b = p<0.01 compared to controls.

Rat neonatal H9c2 myoblasts were used to test the hypothesis that paracrine factors secreted by MSC inhibit caspase-3, a later marker of apoptosis. H9c2 cells were cultured in the presence or absence of CM while under hypoxic conditions for 24 hours to induce apoptosis and then tested for caspase-3 activity (n = 6). As shown in Figure 4, CM was able to significantly (p<0.05) reduce caspase-3 activity to 84.5±5.5% of control levels.

Since Akt [3] and Bad [4] are involved in the apoptotic pathway, the phosphorylation state of these proteins in H9c2 cells was monitored using ELISA (Figure 4. Phospho-Akt (Ser473) and phospho-Bad (Ser112) were significantly reduced by CM after 24 hours to 79.0±6.4% and 60.6±7.9% of control values (p<0.05; n = 6), respectively. In contrast, CM significantly increased phospho-Akt (Thr308) to 147.5±15.3% of control values (p<0.01; n = 12) in H9c2 cells. No changes in total Akt or Bad were observed (data not shown).

### Effect of MCP-1 and PI 3-Kγ inhibitor on Caspase-3 and Akt/Bad Phosphorylation in H9c2 Cells

Since MCP-1 is a major paracrine factor secreted by MSC, we wanted to determine whether changes observed after CM treatment were due to this cytokine. Therefore, H9c2 cells were cultured under hypoxic conditions for 24 hours in either Mesencult (control) or Mesencult containing 4 ng/ml MCP-1 with or without 1 µM phosphoinositol-3-kinase-gamma (PI 3-Kγ) inhibitor for 24 hours (n = 6). PI 3-Kγ is activated by MCP-1 during induction of chemotaxis [5]. A concentration range of 0.001–1.0 µM PI 3Kγ had no effect on caspase-3 activity in control cultures while 20 and 200 µM completely suppressed this activity (data not shown).

**Figure 5:**
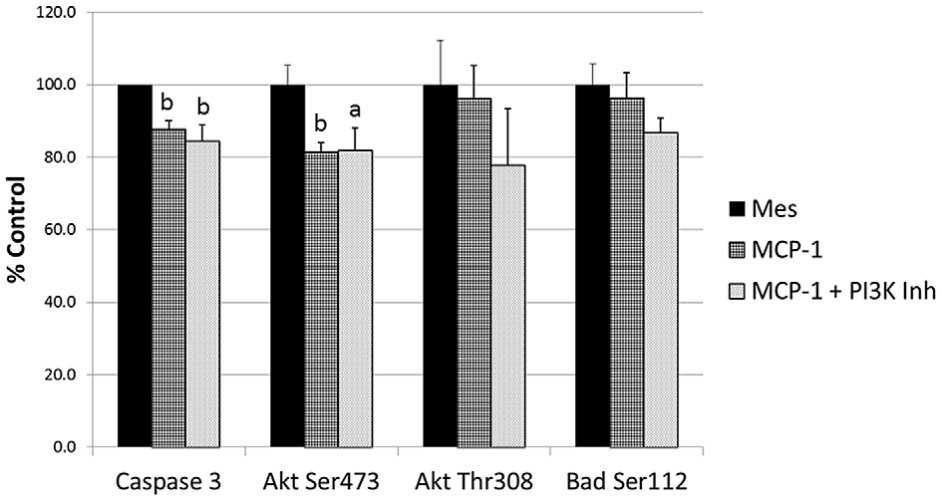
Effect of MCP-1 and PI 3-Kγ inhibitor on caspase-3, Akt and Bad. Changes in caspase-3, phospho-Akt (Ser473), phospho-Akt (Thr308) and phospho-Bad (Ser112) in H9c2 cells treated with Mesencult (Mes), MCP-1 or MCP-1 + PI 3-Kγ inhibitor for 24 hours under hypoxic conditions. Data calculated as a percent of Mesencult (control) treated cultures ± SE. a = p<0.05, b = p<0.01 compared to controls.

**Figure 6:**
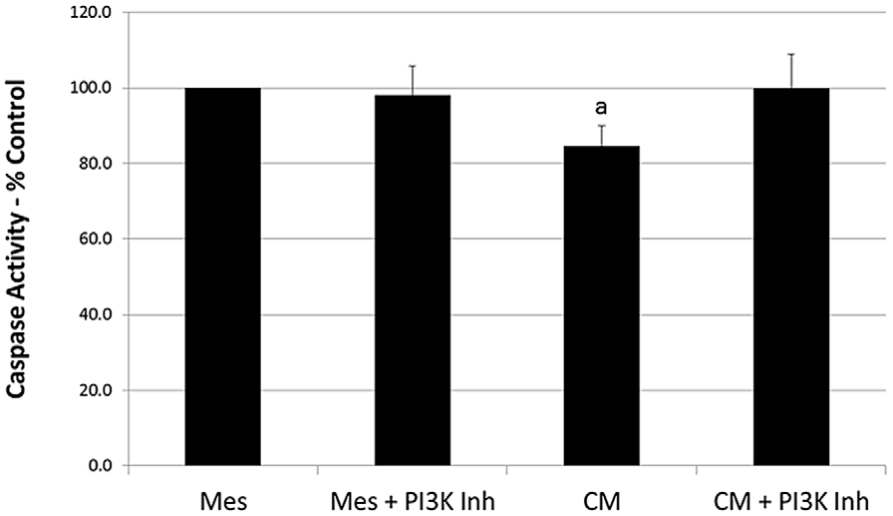
Effect of MSC-conditioned media and PI 3-Kγ inhibitor on caspase-3 activity. H9c2 cells treated for 24 hours under hypoxic conditions. Data calculated as a percent of Mesencult (control) treated cultures ± SE. a = p<0.05 compared to controls.

The addition of MCP-1 resulted in a significant decrease (p<0.01) in caspase-3 activity to 87.7±2.4% similar to that found for CM; however, the PI 3-Kγ inhibitor did not reverse the effect of MCP-1 on caspase-3 (Figure 5). Interestingly, when PI 3-Kγ inhibitor was added to CM, it was able to reverse the effect of CM on caspase-3 while having no effect on control cultures (Figure 6).

MCP-1 treatment significantly reduced (p<0.01) phospho-Akt (Ser473) compared to Mesencult controls (81.4±2.5% of control), while the inclusion of the PI 3-Kγ inhibitor with MCP-1 did not alter its effect (Figure 5). MCP-1 and MCP-1 + PI 3-Kγ inhibitor had no significant effect on phospho-Akt (Thr308) or phospho-Bad (Ser112) compared to Mesencult controls (Figure 5). Total Akt and Bad were unaffected by the treatments as well (data not shown). The decline in phospho-Akt (Ser473) caused by MCP-1 was similar to that found for CM; however, changes in phospho-Akt (Thr308) and phospho-Bad (Ser112) induced by CM did not occur in response to MCP-1 (compare Figures 4 and 5).

### Role of ERK during the Effect of Conditioned Media on Intracellular Signaling in H9c2 Cells

**Figure 7:**
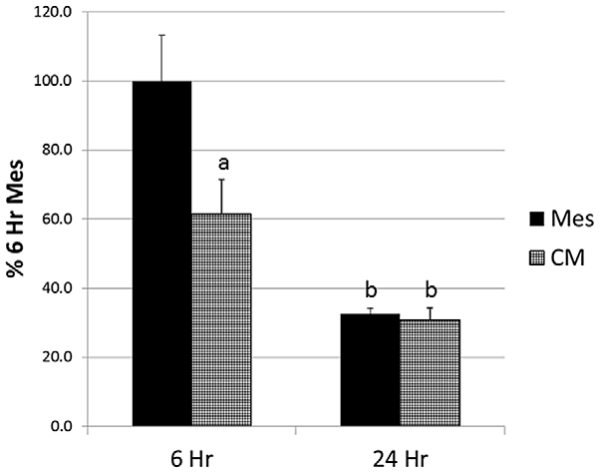
Effect of MSC-conditioned media on phospho-ERK 1/2. H9c2 cells treated for 6 or 24 hours under hypoxic conditions. Data calculated as a percent of the 6-hr Mesencult (control) treated cultures ± SE. a = p<0.05, b = p<0.01 compared to controls.

Since phosphorylated ERK is an important kinase activated during receptor mediated intracellular signaling, we wanted to test the role that ERK might play in the changes occurring in H9c2 cells after CM treatment. Therefore, phospho-ERK 1/2 was monitored by ELISA in H9c2 cells after 6 and 24 hours of treatment with Mesencult or CM under hypoxic conditions (n = 6). As seen in Figure 7, CM significantly reduced the levels of phospho-ERK 1/2 after 6 hours to 61.4±9.9% of control (p<0.05). There was no difference between control and CM treated cells after 24 hours (32.5±1.7% and 30.8±3.5% of the 6 hour control), but the levels of both were significantly lower after 24 hours compared to the 6 hour control (p<0.01).

**Figure 8:**
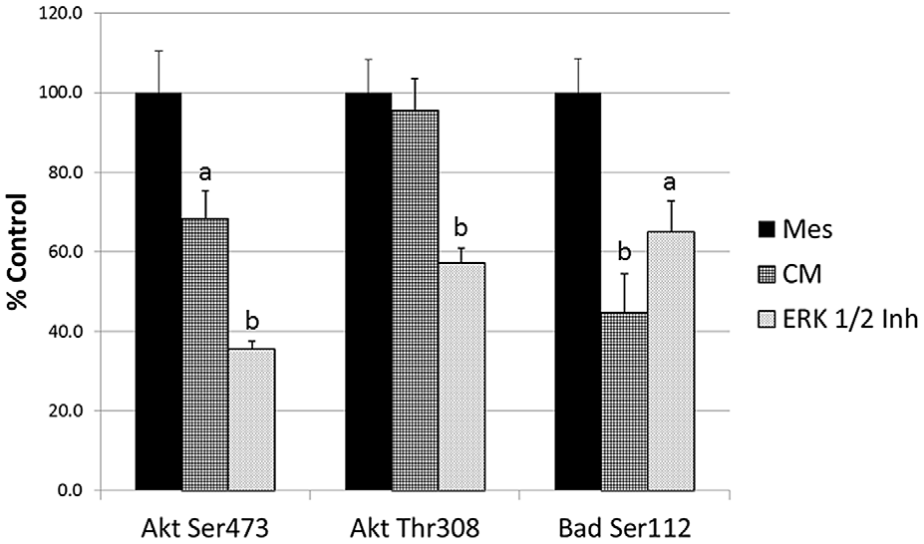
Effect of MSC-conditioned media and ERK 1/2 inhibitor on Akt and Bad phosphorylation. Changes in phospho-Akt (Ser473), phospho-Akt (Thr308) and phospho-Bad (Ser112) in H9c2 cells treated with Mesencult (Mes), conditioned media (CM) or ERK 1/2 inhibitor for 6 hours under hypoxic conditions. Data calculated as a percent of Mesencult (control) treated cultures ± SE. a = p<0.05, b = p<0.01 compared to controls.

Since phospho-ERK 1/2 levels were significantly lower in CM treated cells after 6 hours, we wanted to determine whether this loss of ERK 1/2 activation was responsible for the changes seen in phospho-Akt and phospho-Bad. H9c2 cells were treated with Mesencult, CM, or 30 µM ERK 1/2 inhibitor under hypoxic conditions for 6 hours (n = 6). As shown in Figure 8, CM significantly reduced phospho-Akt (Ser473) to 68.4±6.9% and phospho-Bad (Ser112) to 44.8±9.7% of control values (p<0.05 and 0.01, respectively). ERK 1/2 inhibition resulted in a similar significant reduction of phospho-Akt (Ser473) and phospho-Bad (Ser112) after 6 hours to 35.6±1.9% (p<0.01) and 65.1±7.7% (p<0.05) compared to controls. Phospho-Akt (Thr308) levels were maintained in CM treated cells after 6 hours (91.2±5.1; n = 6); however, ERK 1/2 inhibition resulted in a significant decline in phospho-Akt (Thr308) levels to 46.9±2.0% (p<0.01; n = 6). Comparing the changes at 6 hours (Figure 8) with those at 24 hours (Figure 4), CM caused a similar decline in phospho-Akt (Ser473) and phospho-Bad (Ser112) at both time points. However, the increase in phospho-Akt (Thr308) seen at 24 hours was not yet present at 6 hours.

## Discussion

Our study clearly identifies specific bone marrow-derived MSC secreted paracrine factors that are able to induce angiogenesis, affect cellular migration and attenuate caspase-3. This supports our previous *in vivo* study [1] where we concluded that the cardioprotective effect of intravenous administration of MSC after myocardial infarction was likely due to paracrine secretions from the MSC, a mechanism supported by other investigators [6]–[8]. Unique to our study, we quantified secretion of VEGF, MCP-1, MIG, MIP-1α, and MIP-1β by MSC into the surrounding media, and were able to detect mRNA in MSC for the same factors. Basic FGF, MIP-3β, RANTES, INF-γ, TNF-α, and PDGF were also measured but were not elevated compared to control levels. Singla and McDonald [9] found that human embryonic stem cells released paracrine factors that reduced apoptosis in H9c2 cells and focused on TIMP-1 (tissue inhibitor of metalloproteinase) as an important molecule in this process. Other investigators have identified a number of factors secreted by cord blood derived [10] and embryonic stem cell derived [11] MSC including VEGF and MCP-1 but did not determine their specific biologic effects.

We were able to demonstrate significant biologic activity for the factors secreted by MSC. MSC-conditioned media promoted angiogenesis by CVEC, and the conditioned media components VEGF and MCP-1 have established angiogenic properties [12]–[14]. We demonstrated that both MCP-1 and MIP-1α were able to promote cellular migration of MSC while VEGF inhibited MSC migration. Other investigators have shown that all three factors promote MSC migration[15]–[19], although a few reports were unable to demonstrate an effect of MCP-1 and VEGF[20], [21]. Our result showing a decline in MSC migration after VEGF might be explained by differences in culture conditions, the most notable being our high serum concentration (20% FBS in Mesencult vs. 1% or less in most studies). MSC-conditioned media reduced caspase-3 activity in H9c2 cells, and MCP-1 was able to mimic this pro-survival effect. In addition to promoting monocyte chemotaxis, MCP-1 has been shown to be both pro- and anti-apoptotic in cardiomyocytes [22], [23]. The G-protein coupled receptor for MCP-1, CCR2, can act through Gαi, Gαs or Gαq depending on the cell type [24]. Interestingly, Tarzami and coworkers [23] found that while the stimulation of chemotaxis by MCP-1 in monocytes was dependent upon the activation of Gαi and Gαs, the pro-survival effect of MCP-1 in cardiomyocytes acted independent of these, most likely through the Gαq pathway.

The chemoattractant properties of MCP-1 are mediated through the gamma isoform of PI-3 kinase [5]. However, the reduction in caspase-3 activity by MCP-1 in our study was mediated by an intracellular signaling pathway independent of PI-3 kinase γ since the addition of a specific inhibitor of this enzyme along with MCP-1 did not reduce the protective effect of this chemokine. Interestingly, the addition of the PI-3 kinase γ inhibitor to MSC-conditioned media did prevent its protective effect suggesting that conditioned media contains other pro-survival factors in addition to MCP-1 which may act through PI-3 kinase γ-dependent pathways. These findings are consistent with Singla and coworkers [25] who reported that the PI-3 kinase inhibitor, LY-294002 was able to abolish the protective effect of embryonic stem cell conditioned media in H9c2 cells, although it was not reported whether MCP-1 was elevated in their conditioned media, and LY-294002 is not specific to the gamma isoform of PI-3 kinase. Taken together, these data suggest that multiple paracrine factors in conditioned media, possibly from different cell types within the stem cell population, are responsible for the pro-survival response and exert their effects through both PI-3 kinase dependent and independent pathways.

We found that phospho-Akt (Ser473) was lower in MSC-conditioned media treated cells while phospho-Akt (Thr308) was elevated. MCP-1 also reduced phospho-Akt (Ser473) but had no effect on Thr308; PI-3 kinase γ inhibition concurrent with MCP-1 yielded similar results. These findings suggest that 1) phosphorylation of Akt at Thr308 might be responsible for the pro-survival effect of conditioned media and 2) in addition to its PI-3 kinase independent effect on caspase-3 activity, the protective effect of MCP-1 is not mediated by Akt activation. Activation of PI-3 kinase results in the activation of PDK1 which phosphorylates Akt at the Thr308 site while the process responsible for phosphorylation of the Ser473 site is less clear and may involve mTOR, autophosphorylation, or other mechanisms [26]. Akt is known to be part of a pro-survival pathway; phosphorylation at Thr308 is required for Akt activation and increases its catalytic activity 100 fold while phosphorylation at Ser473 is dependent on phosphorylation at Thr308 and potentiates its activation approximately 10 fold [3].

Since multiple signaling pathways are involved, we also studied ERK 1/2 and found that phospho-ERK 1/2 was reduced in H9c2 cells following 6 hour treatment with CM, suggesting that inhibition of ERK was part of the overall response of these cells to CM. Phosphorylation of ERK 1/2 is involved in doxorubicin-mediated cell death in H9c2 cells [27], and oxidative stress in these cells promotes both cell death and ERK activation [28]. Whether ERK is involved in proliferation or cell death depends on the cell type and signaling molecules present [29]. Our results also show that direct ERK 1/2 inhibition reduced phosphorylation at both Ser473 and Thr308 suggesting that ERK regulation after CM treatment may be involved in the reduction of Ser473 but not the increase in Thr308. Interestingly, MSC-conditioned media maintained phosphorylation at Thr308 after 6 hours and increased it after 24 hours. Thus, although ERK 1/2 signaling is reduced by conditioned media, some other factor in the media counteracts the effect of ERK inhibition on Thr308 phosphorylation causing it to rise.

Another potential target in the apoptotic signaling pathway that we explored was Bad phosphorylation. Bad is also anti-apoptotic when phosphorylated [3], [4] but its control pathway is very complex. Bad is pro-apoptotic and binds via its BH3 domain to anti-apoptotic Bcl-2/Bcl-X_L_, inhibiting its function. Bad can be phosphorylated at a number of serine sites (Ser112, 128, 136, 155, 170), preventing it from binding to Bcl-2/Bcl-X_L_ and inhibiting this anti-apoptotic pathway [30]–[34]. Bad is phosphorylated via a number of signaling systems; for example, PKA [35], RSK1 [33] and ERK (MAPK) [30] can phosphorylate Bad at Ser112, Akt (PKB) phosphorylates Bad at Ser136 [31], and PKA, RSK1 and survival factor are involved in phosphorylation at Ser155. Phosphorylation at any of these sites promotes its binding and subsequent sequestration by 14-3-3 proteins preventing binding to Bcl-2/Bcl-X_L_. Dephosphorylation at both Ser112 and Ser136 is required for release from 14-3-3 [30], [31]. We found that both MSC conditioned media and ERK 1/2 inhibition reduced phospho-Bad (Ser112) while MCP-1 alone had no effect. As discussed above, other factors are most likely present in the MSC-conditioned media that control apoptosis and these might act through phosphorylation of Akt at Thr308. It is not immediately clear why Akt (Ser473) and Bad (Ser112) were reduced, since this would tend to promote and not inhibit apoptosis; however, other Bad phosphorylation sites or downstream effectors may be involved in the pro-survival effect of MSC-conditioned media.

In summary, bone marrow MSC secretes factors that act in a paracrine manner to promote angiogenesis, alter cell migration and inhibit apoptosis. Both MCP-1 and MIP-1α were able to promote cellular migration of MSC and MCP-1 displayed a protective effect by reducing caspase-3 activity in H9c2 cells. The overall protective effect of CM was demonstrated to include PI 3-kinase and the phosphorlylation of Akt (Thr308), but the MCP-1 effect was independent of PI 3-kinase and Thr308 phosphorylation. CM also caused a reduction in ERK 1/2 activity that was unrelated to the increase in Thr308 phosphorylation. It is likely that multiple pro-survival factors in addition to MCP-1 are secreted by MSC which act on several pathways. Further study will help delineate the specific pathways used by MCP-1 and the other identified factors and whether they contribute to the beneficial effects of stem cell therapy following tissue damage after myocardial infarction.
